# Management of an elderly patient with nonsyndromic *TGFBR1*‐related aortopathy: A case report

**DOI:** 10.1002/ccr3.9317

**Published:** 2024-08-08

**Authors:** Hitomi Aono‐Setoguchi, Hiroki Yagi, Nana Akiyama, Norifumi Takeda, Masahiko Ando, Haruo Yamauchi, Issei Komuro, Norihiko Takeda

**Affiliations:** ^1^ Department of Cardiovascular Medicine, Graduate School of Medicine University of Tokyo Tokyo Japan; ^2^ Marfan Syndrome Center University of Tokyo Hospital Tokyo Japan; ^3^ Department of Genomic Medicine University of Tokyo Hospital Tokyo Japan; ^4^ Department of Cardiovascular Surgery University of Tokyo Hospital Tokyo Japan; ^5^ Department of Frontier Cardiovascular Science, Graduate School of Medicine University of Tokyo Tokyo Japan; ^6^ International University of Health and Welfare Tokyo Japan

**Keywords:** hereditary aortic disease, Loeys–Dietz syndrome, next‐generation sequencing, nonsyndromic *TGFBR1*‐related aortopathy

## Abstract

**Key Clinical Message:**

Genetic variants associated with hereditary TAAD may contribute to nonsyndromic TAAD. We present the case of a 72‐year‐old man with nonsyndromic TAAD undergoing prophylactic surgery after a gene panel test revealed a pathogenic variant in *TGFBR1*, but the indication for genetic testing in such elderly‐onset cases still warrants further discussion.

**Abstract:**

Hereditary thoracic aortic aneurysm and dissection (TAAD) is a *serious* clinical condition resulting in a fatal outcome. Recently, variants in causative genes for syndromic hereditary TAAD, such as Marfan syndrome and Loeys–Dietz syndrome (LDS), have been reported to predispose to the development of nonsyndromic TAAD; however, genetic testing for patients with elderly‐onset nonsyndromic TAAD warrants further discussion. We present a 72‐year‐old nonsyndromic Japanese man with moderate‐sized aortic annulus ectasia (AAE) with moderate aortic regurgitation and ascending to distal arch aortic dilatation (maximum diameter: 46 mm). He had been treated for hypertension and dyslipidemia for 7 years, and his eldest son had AAE at 33 years old and type A aortic dissection at 43 years old. Surgical repair was considered a treatment option because the patient potentially had a nonsyndromic hereditary aortic disease, and genetic panel testing for TAAD identified a pathogenic missense variant in *TGFBR1* (c.934G > A, p.[Gly312Ser]), previously reported in patients with LDS type 1. He was diagnosed with nonsyndromic *TGFBR1*‐related aortopathy and underwent prophylactic surgery using a modified Bentall operation and total arch replacement with open stent graft implantation. Genetic testing was useful in guiding the treatment strategy, but further analysis is warranted to establish the clinical value in the treatment plan for patients with elderly‐onset nonsyndromic TAAD.

## 1 INTRODUCTION

Hereditary thoracic aortic aneurysm and dissection (TAAD) can be fatal early in life if patients do not receive appropriate care. Genetic testing for known causative genes associated with hereditary TAAD, such as the 11 “Category A” genes defined by the ClinGen Aortopathy Working Group,^1^ could help guide medical decisions for prevention, diagnosis, and treatment.^1^ Since 2016, genetic testing for aortopathy to guide precision medicine has been covered by health insurance in Japan, and next‐generation sequencing‐based gene panel tests, including *FBN1* (OMIM*134797), *TGFBR1* (OMIM*190181), *TGFBR2* (OMIM*190182), *SMAD3* (OMIM*603109), *TGFB2* (OMIM*190220), *TGFB3* (OMIM*190230), *SMAD2* (OMIM*601366), *COL3A1* (OMIM*120180), *ACTA2* (OMIM*102620), *MYH11* (OMIM*160745), *MYLK* (OMIM*600922), and *PRKG1* (OMIM*176894), have become widely available with appropriate genetic counseling.^2^

Loeys–Dietz syndrome (LDS) is an autosomal dominant genetic disorder closely related to Marfan syndrome (MFS). LDS can lead to aortic aneurysm and dissection but not ectopia lentis and is characterized by a triad of arterial tortuosity and aneurysm, hypertelorism, and bifid/broad uvula or cleft palate. LDS is caused by an abnormality in transforming growth factor‐β (TGF‐β) signaling pathway‐related genes,^3^, ^4^ and fatal aortic dissection (AD) and rupture often occur at a younger age and with smaller vessel diameters than MFS, particularly in Type 1–3 LDS (*TGFBR1/2*: LDS1/2; *SMAD3*: LDS3). However, clinical features overlap with MFS, and the diagnostic and treatment strategies are often established based on genetic testing results.^5^

Because genetic testing for aortopathy is widely used, phenotypes associated with *TGBFR1* and *TGFBR2* pathogenic variants include milder forms of LDS and nonsyndromic forms of TAAD with decreased penetrance,^3^, ^4^, ^6^, ^7^ which further enhances the clinical value of genetic testing to determine the treatment strategy. Since April 2018, we have performed a gene panel test for hereditary TAAD at our MFS center,^8^ and we have encountered some patients with a pathogenic or likely pathogenic variant in TGF‐β‐related genes without typical extra arterial features commonly observed in patients with LDS. Here, we present a case of an elderly Japanese man with nonsyndromic moderate‐sized TAA with multiple atherosclerotic risk factors and a family history of type A AD in his son. The patient had a pathogenic variant of *TGFBR1* and was surgically treated using modified Bentall operation and total arch replacement with open stent graft implantation.

## 2 MATERIALS AND METHODS

### 2.1 Genetic analyses of hereditary TAAD‐related genes

The Institutional Ethics Committee of the University of Tokyo Hospital approved the genetic testing for hereditary TAAD (reference No. G‐1538), and the patients provided written informed consent. The hybridization capture‐based gene panel test for hereditary TAAD, containing *FBN1*, *TGFBR1*, *TGFBR2*, *TGFB2*, *TGFB3*, *SMAD2*, *SMAD3*, *ACTA2*, *MYH11*, *MYLK*, and *COL3A1* genes, was provided by the Kazusa DNA Research Institute (Chiba, Japan) under the coverage of Japanese health insurance.^8^, ^9^

### 2.2 Human aortic tissue samples

The Institutional Ethics Committee of the University of Tokyo Hospital approved the study of human aortic tissue (reference No. 2233‐7). During elective prophylactic surgery, aortic aneurysm tissue from the Valsalva sinus to the ascending aorta was obtained from the proband (MT 40) (II‐1). We obtained a control aortic tissue from a non‐familial TAA in a 69‐year‐old patient (MT 89).

### 2.3 Histological analysis and immunohistochemical staining

Aortic tissues were fixed in 10% formalin, embedded in paraffin, and sectioned at 5‐μm thickness. Four serial sections were stained using Elastica van Gieson and Masson's trichrome, antiphosphorylated SMAD2 (Ser465/467) antibody (Cell Signaling Technology, #3108), and antiphosphorylated ERK1/2 (Thr202/Tyr 204) antibody (Cell Signaling Technology, #9101) by using a VECTASTAIN ABC Kit (Vector Laboratories, PK‐4001) and 3,3′‐diaminobenzidine tetrahydrochloride (Vector Laboratories, SK‐4100).

## 3 CASE REPORT

**FIGURE 1:**
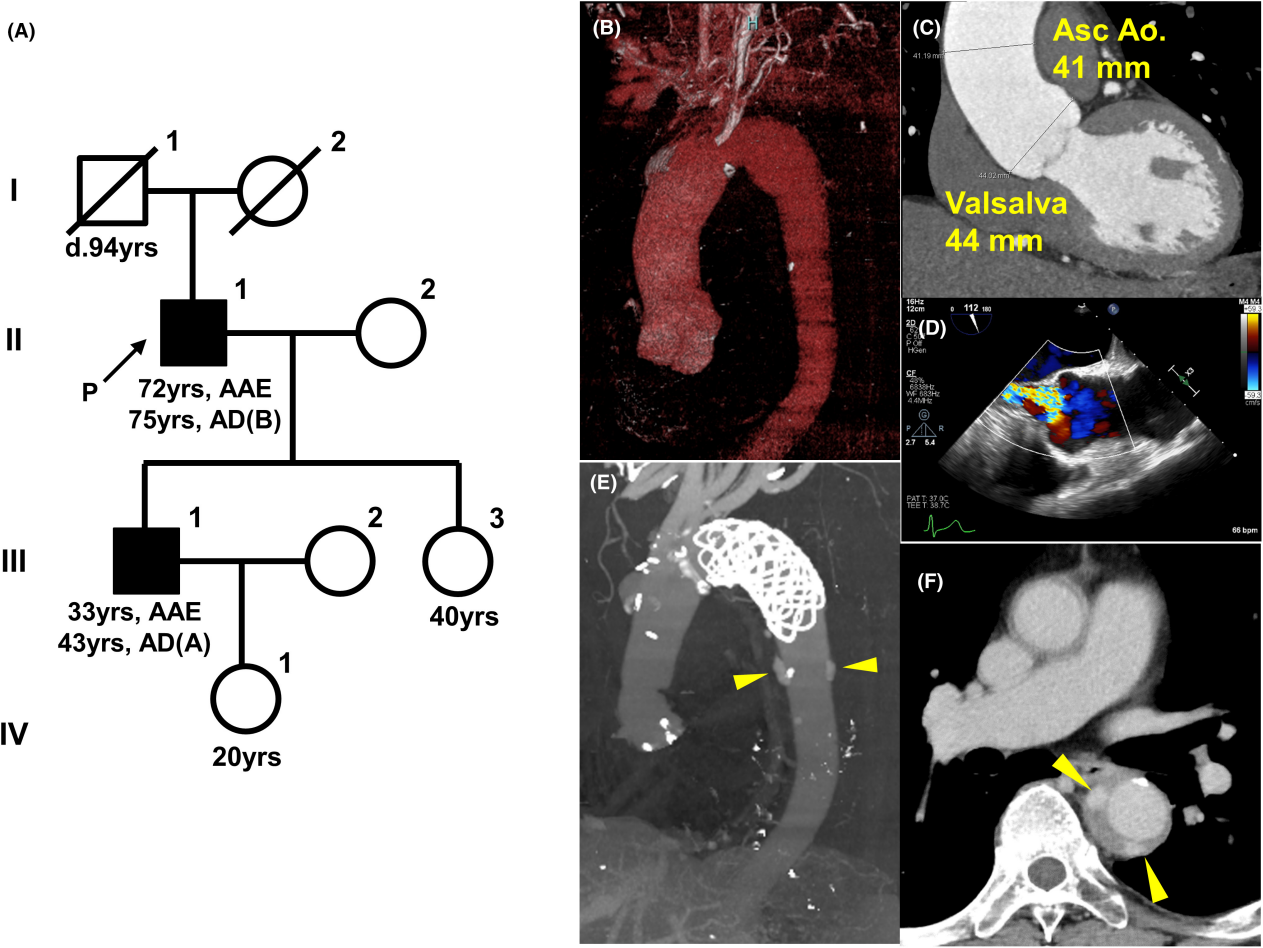
A case of a Japanese patient with nonsyndromic LDS presenting with aortic disease. (A) Pedigree in this proband (II‐1). A black symbol indicates that the patient has thoracic aortic aneurysm and dissection, and “d” indicates age at death. Square, male; circle, female; P and arrow, proband; slash line, died; AAE, aortic annulus ectasia; AD, aortic dissection. (B, C) Contrast‐enhanced computed tomography (3D and sagittal cross‐section) showing the thoracic aneurysm from the Valsalva sinus to the distal aortic arch. The maximum diameter of the ascending aorta, distal arch aorta, and Valsalva sinus was 41, 46, and 44 mm, respectively. (D) Transesophageal echocardiogram reveals the aneurysm from the Valsalva sinus to the ascending aorta and moderate aortic insufficiency. (E, F) Acute type B AD extending from the distal end of the open stent grafting to the iliac artery, with multiple ulcer‐like projections and a retrograde false lumen on contrast‐enhanced computed tomography (3D and axial section). Yellow heads indicate ulcer‐like projections.

A 72‐year‐old male patient was referred to our hospital due to palpitations. He was diagnosed with hypertension (HT) and dyslipidemia (DL) at 65 years old and prescribed amlodipine (5 mg), irbesartan (200 mg), carvedilol (5 mg), and atorvastatin (10 mg) by a local physician. His eldest son had aortic annulus ectasia (AAE) and type A AD at 33 and 43 years old, respectively (Figure 1A). The patient was of normal build (body mass index, 24.9), with a height of 163.7 cm, and weight of 66.7 kg. On auscultation, a third‐degree Levine diastolic reflux murmur was heard, and transthoracic echocardiography revealed a normal left ventricular ejection fraction of 52%, end‐diastolic diameter of 56 mm, end‐systolic diameter of 41 mm, and moderate AAE (44 mm, *Z* score of 3.25) with right coronary cusp bending causing moderate aortic regurgitation. Computed tomography (CT) identified moderate aortic dilatation from the Valsalva sinus to the distal aortic arch ranging from 41 to 46 mm (Figure 1B–D). He had mild scoliosis (Cobb angle, 18°) without any physical findings suggestive of connective tissue disease (CTD), including widely spaced eyes (hypertelorism), bifid uvula, or ectopia lentis. However, surgical repair was considered as a treatment option because of possible nonsyndromic hereditary aortic disease.

A comprehensive genetic panel analysis was performed to guide the treatment strategy and identified a missense variant in exon 5 of *TGFBR1* (c.934G > A, p.[Gly312Ser]; NM_004612.3). The variant was reported in the dbSNP (rs760079636) but not in the minor allele frequency. ExAC reported an allele frequency of 0.00000824 and Human gene mutation database had two literature reports on the variant.^10^, ^11^, ^12^, ^13^, ^14^ In silico pathogenicity analysis of the variant predicted Mendelian clinically applicable pathogenicity (M‐CAP v1.4) and combined annotation‐dependent depletion (CADD v1.7) scores of 0.69 (possibly pathogenic) and 32.0 (highly pathogenic), respectively. Based on the ACMG guidelines,^15^ this variant was classified as pathogenic (PM1, PM2, PM6, PP3, and PP5), and he was diagnosed with nonsyndromic *TGFBR1*‐related TAAD or LDS1, and TAA may have developed due to atherosclerotic risk factors and genetic predisposition. Other family members, including the eldest son (III‐A), did not have the opportunity to visit our center and did not receive genetic diagnosis at the present time.

**FIGURE 2:**
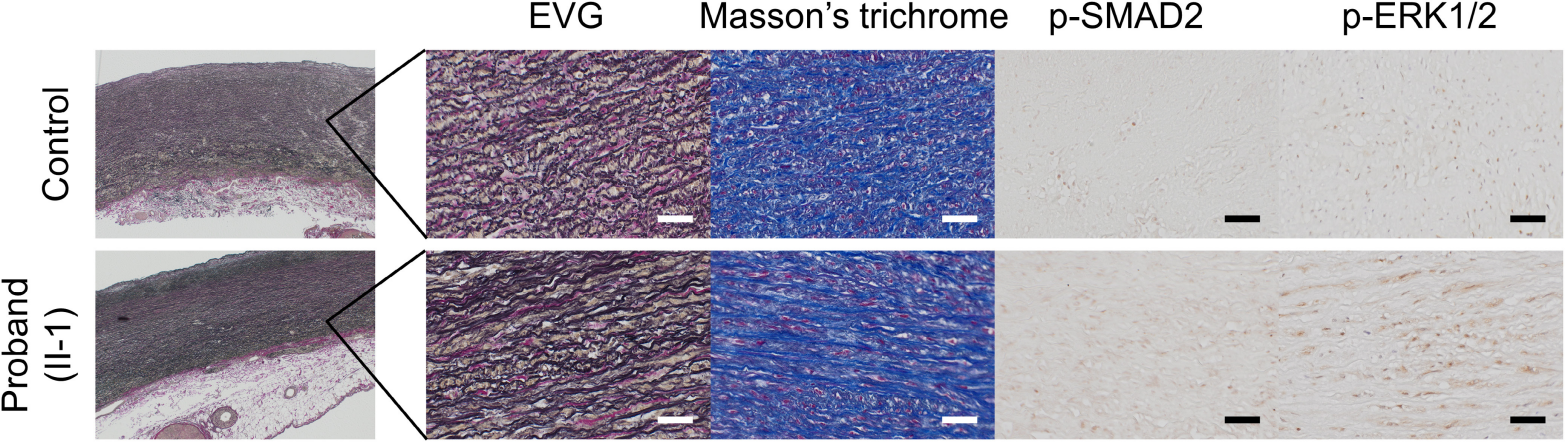
Histological analyses. Histological analyses of the surgically dissected aortic root from the proband (II‐1) and a control from a non‐familial thoracic aortic aneurysm in a 69‐year‐old patient. Elastica van Gieson, Masson's trichrome, and immunohistochemical staining against phosphorylated SMAD2 (p‐SMAD2) and phosphorylated ERK1/2 (p‐ERK1/2) are shown. Scale bar: 50 μm.

At 73 years old, he underwent prophylactic surgery for aortic aneurysm by using a modified Bentall operation and total arch replacement with open stent graft implantation. The aortic root wall showed a similar degree of elastic fiber degeneration and collagen deposition compared with the control aortic specimen from a non‐familial TAA patient (Figure 2). However, compared with the control, the phosphorylation levels of SMAD2 and ERK1/2 in the media were significantly upregulated (Figure 2), indicating that the activated TGF‐β signaling pathways may have played essential roles in promoting TAA formation in this patient. At 75 years old, he developed acute type B AD extending from the distal end of the open stent grafting to the iliac artery, with multiple ulcer‐like projections and a retrograde false lumen (Figure 1E,F). His condition was managed conservatively and remained substantially stable during follow‐up.

## 4 DISCUSSION

Here, we report a case of an elderly Japanese patient with nonsyndromic hereditary TAAD whose gene panel testing identified a *TGFBR1* pathogenic missense variant, which helped guide the treatment strategy. *TGFBR1* is the causative gene for LDS1, one of the most common inherited and aggressive forms of syndromic hereditary TAAD, but has recently been reported to cause nonsyndromic hereditary and slowly progressive TAAD.^3^, ^4^, ^6^, ^7^ Thus, the test must be carefully considered, discussed, and weighed from various clinical points of view.

c.934G > A in *TGFBR1* results in a nonconservative amino acid change (p.[Gly312Ser]) in the protein kinase domain and is found in several patients and families with LDS and nonsyndromic TAAD.^10^, ^11^, ^12^, ^13^, ^14^ Thus, this variant is highly associated with hereditary aortic disease; however, individuals with c.934G > A in *TGFBR1* were free of aortic events until the age of 70 years.^16^ Generally, the presence of extraaortic features (hypertelorism, cervical arterial tortuosity, and wide scars) has been linked to an increased aortic risk in patients with LDS1/2, and those with syndromic LDS require more careful management.^4^, ^7^ Although our elderly patient had nonsyndromic and moderate‐sized TAA, his son had a history of type A AD, and the genetic testing result guided the prophylactic surgery for hereditary TAA to prevent life‐threatening type A AD. Considering a diagnosis of CTD and recommending surgery were challenging in this elderly patient with nonsyndromic and moderate‐sized TAA. Because of his age, he underwent one‐stage TAA repair combined with an open stent graft; however, the indications and long‐term results of the open stent graft for patients with CTD remain debatable. He developed type B AD 2 years postoperatively.

The underlying mechanism of the c.934G > A variant in *TGFBR1* is not clearly understood. LDS1/2 is caused mainly by missense loss‐of‐function variants of *TGFBR1/2*,^17^, ^18^ but overactive TGF‐β signaling has been demonstrated in the aortic wall of patients with LDS and *Tgfbr1* ^*M318R/+*^ and *Tgfbr2* ^*G357W/+*^ knock‐in LDS mice (referred to as the TGF‐β paradox).^19^ In the ascending aorta, cardiac neural crest (CNC) and secondary heart field (SHF) cells of different origins are found separately in the intima and adventitia, respectively, whereas these cells are intermingled in the aortic root.^20^ SHF‐derived vSMCs showed increased TGF‐β1 ligand secretion but poor responsiveness to TGF‐β stimulation and contractility, whereas CNC‐derived vSMCs did not show increased TGF‐β1 ligand secretion but preserved responsiveness to TGF‐β stimulation and contractility.^21^, ^22^ Although TGF‐β activity was not increased in individual CNC‐ and SHF‐derived vSMCs in the in vitro environment, TGF‐β1 ligand secreted by SHF‐derived vSMCs may have acted only on CNC‐derived vSMCs at the aortic root in the in vivo environment to activate the TGF‐β signaling pathway, and the different nature of these lineage‐specific vSMCs would cause discrepancies between the in vivo and in vitro analyses of TGF‐β signaling. In this case, extended histological examination revealed increased phospho‐SMAD2 levels in the aortic root wall, indicating activated TGF‐β signaling in vivo (Figure 2). However, the aortic root (AAE) and the ascending to distal aortic arch were enlarged, which may be influenced by coexisting multiple atherosclerotic risk factors, such as HT and DL, and atherosclerosis has a deleterious effect on survival in *Fbn1* ^*C1041G/+*^ MFS mice.^23^ Appropriate treatment with β‐blockers and angiotensin receptor blockers and prophylactic aortic surgery improves the long‐term prognosis of patients with syndromic hereditary TAAD,^5^ but early intervention against lifestyle‐related diseases may also be crucial in the management of nonsyndromic hereditary TAAD. Thus, further clarification of *TGFBR1*‐related aortopathy and the impact of lifestyle‐related disorders is required.

We report the case of an elderly patient with suspected hereditary TAAD based on family history whose genetic testing led to the diagnosis of elderly‐onset nonsyndromic *TGFBR1*‐related aortopathy complicated by HT and DL. Genetic involvement is rare suspected in TAAD patients over 70 years of age. We retrospectively analyzed 252 cases in which panel analysis of candidate genes for HTAAD was performed at our center since 2018, and only two patients (0.79%), including the present case, were diagnosed with HTAAD at the age of 70 years or older. With the widespread use of genetic testing, variants in causative genes for syndromic hereditary TAAD predispose the patient to develop nonsyndromic TAAD, and guidelines for genetic analysis and management strategy should be improved.^24^ Although genetic testing for patients with elderly‐onset nonsyndromic TAAD with multiple atherosclerotic factors warrants further discussion, comprehensive genetic testing may be useful in developing treatment planning in certain cases with a family history of TAAD.

## AUTHOR CONTRIBUTIONS

**Hitomi Aono‐Setoguchi:** Conceptualization; project administration; writing – original draft. **Hiroki Yagi:** Conceptualization; funding acquisition; project administration; resources; writing – original draft; writing – review and editing. **Nana Akiyama:** Conceptualization; project administration; resources; writing – review and editing. **Norifumi Takeda:** Conceptualization; project administration; resources; writing – original draft; writing – review and editing. **Masahiko Ando:** Conceptualization; project administration; resources; writing – review and editing. **Haruo Yamauchi:** Conceptualization; project administration; resources; writing – review and editing. **Issei Komuro:** Project administration; writing – review and editing. **Norihiko Takeda:** Conceptualization; project administration; writing – review and editing.

## FUNDING INFORMATION

This study was not funded.

## CONFLICT OF INTEREST STATEMENT

The authors have no conflict of interest to declare.

## CONSENT

We confirm that written patient consent was signed and obtained in accordance with the journal's patient consent policy.

## Data Availability

The data supporting the findings of this study are available from the corresponding author upon reasonable request.
